# Majority of drug-related problems identified during medication review are not associated with STOPP/START criteria

**DOI:** 10.1007/s00228-015-1908-x

**Published:** 2015-08-07

**Authors:** Sanne Verdoorn, Henk-Frans Kwint, Adrianne Faber, Jacobijn Gussekloo, Marcel L Bouvy

**Affiliations:** Division of Pharmacoepidemiology & Clinical Pharmacology, Utrecht Institute for Pharmaceutical Sciences (UIPS), 3584 CG Utrecht, The Netherlands; SIR Institute for Pharmacy Practice and Policy, 2331 JE Leiden, The Netherlands; Department of Public Health and Primary Care, Leiden University Medical Center, 2333 ZD Leiden, The Netherlands

**Keywords:** Medication review, Drug-related problems, STOPP/START criteria, Inappropriate prescribing, Elderly, Primary care

## Abstract

**Purpose:**

STOPP and START criteria identify potential inappropriate prescribing and potential prescribing omissions. It is unknown whether STOPP/START criteria identify all drug-related problems. This study aims to determine to what extent STOPP/START correspond to drug-related problems (DRPs) identified during a full clinical medication review.

**Methods:**

In 13 Dutch community pharmacies, 457 community-dwelling patients aged ≥65 years and using ≥5 drugs, received a full clinical medication review. Community pharmacists identified potential DRPs and recommendations by implicit criteria. After completion, all identified DRPs and recommendations were compared with STOPP and START criteria by investigators.

**Results:**

The total number of potential DRPs identified by community pharmacists was 1656 in 457 patients (mean 3.6 per patient). Eighty-one percent of DRPs were not associated with STOPP/START criteria. The percentage of START criteria present in identified DRPs was higher than the percentage of STOPP criteria (13 vs. 5.7 %, *p* < 0.01).

The implementation rate for recommendations associated with STOPP criteria was higher compared to recommendations associated with START criteria (56 vs. 39 %, *p* < 0.01). Both implementation rates of STOPP and START recommendations were lower compared to recommendations not associated with STOPP/START criteria (66 %, *p* = 0.047 and *p* < 0.001, respectively).

**Conclusions:**

This study shows that the majority of drug-related problems of community-dwelling older patients was not associated with STOPP/START criteria. These findings suggest that application of STOPP/START criteria in full clinical medication review should preferably be combined with implicit criteria.

## Introduction

Polypharmacy and inappropriate medication use by older people increase the risk of adverse drug reactions [1]. Inappropriate medications are defined as medications for which the potential risk outweighs the potential benefit [2]. Next to inappropriate medications, older patients with polypharmacy may also be susceptible to under-prescribing. Under-prescribing of medications refers to the omission of a drug when there is a clear indication and no contra-indication [3].

Several tools are available to evaluate inappropriate medication and prescribing omissions in older patients, including implicit and explicit criteria. Implicit criteria, like the Medication Appropriateness Index (MAI), consist of a structural assessment of the patient’s medicines. They may rely on expert professional judgment for their application. Although implicit criteria have demonstrated their usefulness in detecting drug-related problems in several studies, the application may be rather time-consuming in practice [4–12].

Explicit criteria consist of lists of inappropriate medications and prescribing omissions in the elderly. The first explicit criteria published for potentially inappropriate medications were the Beers-criteria [2, 13–15]. These are the most widely cited criteria for inappropriate medications, but the applicability outside the USA is limited due to differences in types of drugs and guidelines [16–18]. In 2008, two sets of European-based criteria (STOPP and START) were formulated to address the perceived deficiencies of Beers’ criteria and prescribing omissions as well [19]. Screening Tool of Older Persons Prescriptions (STOPP) contains a list of 65 potentially inappropriate medications or medication classes. Screening Tool to Alert doctors to Right Treatment (START) lists 22 potential prescribing omissions (PPOs) in patients with particular medical conditions [19].

Studies in older patients showed that STOPP criteria were more sensitive than Beers criteria in identifying potentially inappropriate medications [20–22]. However, there are no studies that compared the use of these explicit criteria with implicit criteria in the detection of drug-related problems. Therefore, the aim of our study was to determine the number and types of STOPP/START criteria present in identified drug-related problems (DRPs) and recommendations found by medication review with implicit criteria.

## Methods

### Study procedures and population

In 13 Dutch community pharmacies, a list of all community-dwelling patients aged 65 years and older, using at least five oral prescription drugs in 2011, was compiled using the pharmacy information system. From this list, the pharmacists took a convenience sample of patients to invite for a medication review. Pharmacies were located in both urban regions in the south-west of the Netherlands. Pharmacists received complete medical data from the general practitioners (GPs), including diagnoses and laboratory values, after agreement of the patient.

### Ethics

Medication reviews are provided as an enhanced service for older people with polypharmacy by pharmacists and GPs in the Netherlands. In order to protect the patient’s privacy, all data were anonymised by the community pharmacists using a randomly assigned unique number. The researchers only received the anonymised written care plans of the medication reviews. In addition, they received anonymised drug dispensing records for each patient. Because this study was performed retrospectively and used anonymised patient data, no ethical approval was required according to current Dutch guidelines.

### Medication review

The patient’s community pharmacist interviewed the patient about his drugs at home or in the pharmacy. Patient’s concerns and experiences regarding drug therapy (in particular perception of the effectiveness and potential adverse effects), adherence issues, practical problems, understanding of their medication regimen and possible use of OTC (over the counter) medication were addressed during this interview. A pharmaceutical care plan was proposed by the community pharmacist using both the patient’s medication records from the pharmacy, general practitioners (GPs) medical records and the data from the patient interview. Potential DRPs and associated recommendations were identified by implicit criteria based on a structural assessment of indication, effectiveness, safety and compliance by Hepler and Strand [7]. Recommendations were implemented after agreement between both the community pharmacist, the general practitioner and the patient. Follow-up of implemented recommendations was monitored by the community pharmacists.

Participating community pharmacists had experience in performing medication reviews. Therefore, they received an accredited training course in medication review. The course educated in clinical guidelines, communication skills, identifying DRPs and designing pharmaceutical care plans. In addition, pharmacists participated in monthly web conference sessions moderated by a medication review expert. During these sessions, case studies and treatment guidelines were discussed. Moreover, all pharmacists were observed and received feedback on one medication review session.

### Data classification

Drugs were classified using the Anatomical Therapeutic Chemical (ATC) Classification System (11th edition, 2008) formulated by the World Health Organization Collaborating Centre for Drug Statistics Methodology. Potential DRPs and recommendations were classified according to the D.O.C.U.M.E.N.T. system by the community pharmacists [12, 23–25].

### Application of START-STOPP criteria

After completion of the medication reviews, the anonymised results were sent to the investigators. The database with the results consisted of registered DRP’s, ATC codes of the drugs associated with the DRPs and interventions, recommendations and free text boxes with reasons to change a drug (e.g. start ACE inhibitor because of heart failure). Based on these results, STOPP and START criteria were retrospectively and independently applied by two investigators (H.K. and S.V.). Differences were discussed until consensus was reached. A 10 % sample of the DRPs was taken and also reviewed by two other investigators (A.F. and M.B.). For the application of the STOPP/START criteria, the first version of the STOPP/START criteria was used [19]. Two adaptations of the original criteria were made to make the criteria fit the current Dutch guideline [8]. The STOPP criteria: “aspirin or NSAIDs without a proton pump inhibitor” and “opiates without laxatives”, were modified to START criteria [8].

### Outcome measurements

The primary outcomes were number, type and implementation rate of STOPP and START criteria applicable to identified DRPs and associated recommendations during medication review. The implementation rate was defined as the percentage of recommendations that was fully or partly implemented according to the community pharmacist.

### Statistical analysis

All implementation rates of recommendations were analysed on an intention-to-treat basis. Descriptive statistics were used for basic characteristics. Pearson chi-square tests were used for each categorical variable. A *p* value <0.05 was considered statistically significant. All data were analysed using Microsoft Access and Excel 2010 (Microsoft Corporation, Redmond, WA, USA) and SPSS version 20.0 (SPSS Inc., Chicago, IL, USA).

## Results

### Demographics

Twenty-one community pharmacists in 13 pharmacies collaborated with 65 GPs in this study. The pharmacists conducted 533 patient interviews. Clinical medication reviews were performed for 461 of 533 patients. Seventy-two patients were excluded because their medication reviews were not fully completed. Of 461, another four patients were excluded during follow-up because of death or hospital admission. Finally, 457 patients were included for analysis. The median age was 77 years (interquartile range 73–81), and 60 % were women (Table 1). The most commonly prescribed drug classes were “antithrombotic agents” (69 %) and “agents acting on the renin-angiotensin system” (68 %).

**Table 1:** Baseline characteristics of participants (≥65 years and ≥5 drugs) *n* = 457

Socio demographic		
Female (%)	60	
Age (year, median, interquartile range)	77	(73–81)
Number of prescribed drugs (mean per patient, SD)	8.7	(3.2)
Most prescribed drug classes (ATC)		
Antithrombotic agents (B01A)	69 %	
Agents acting on the Renin-Angiotensin System (C09)	68 %	
Lipid Modifying agents (C10A)	59 %	
Beta blocking agents (C07A)	57 %	
Drugs for peptic ulcer and GORD (A02B)	57 %	
Drugs used in diabetes (A10)	33 %	
High-ceiling diuretics (C03C)	31 %	
Calcium channel blockers (C08C)	27 %	
Low-ceiling diuretics (C03A, C03B, C03E)	24 %	
Drugs for obstructive airway diseases (R03)	22 %	
Benzodiazepine derivatives (N05BA, N05CD)	22 %	

*ATC* Anatomical Therapeutic Chemical Classification System

### STOPP and START criteria among identified DRPs

A total of 1656 potential DRPs were identified (mean 3.6 per patient) (Table 2). Eighty-one percent of DRPs were not associated with either STOPP or START criteria. The percentage of START criteria present in identified DRPs was higher than the percentage of STOPP criteria (13 vs. 5.7 %, *p* = 0.001).

**Table 2:** Classification of identified DRPs by STOPP and START criteria

DRP type and subtype		Not identified by STOPP/START		Identified by STOPP		Identified by START	
	*N*	*N*	(%)^b^	*N*	(%)^b^	*N*	(%)^b^
D(rug selection)	**303**	**239**	**(79)**	**59**	**(19)**	**5**	**(2)**
Duplication	20	3^a^	(15)	17	(85)	–	–
Drug interaction	11	10	(91)	1	(9)	–	–
Contra-indication apparent	16	7	(44)	7	(44)	2	(12)
No indication apparent	256	219	(86)	34	(13)	3	(1)
O(ver or underdose)	**200**	**199**	**(99)**	**1**	**(1)**	–	–
Prescribed dosage too high	64	63	(99)	1	(1)	–	–
Prescribed dosage too low	52	52	(100)	–	–	–	–
Incorrect or unclear dosing instructions	84	84	(100)	–	–	–	–
C(ompliance)	**142**	**139**	**(98)**	**1**	**(1)**	**2**	**(1)**
Taking too little	72	69	(96)	1	(1)	2	(3)
Taking too much	8	8	(100)	–	–	–	–
Difficulty using dosage form	62	62	(100)	–	–	–	–
U(ndertreated)	**507**	**289**	**(57)**	**13**	**(3)**	**205**	**(40)**
Condition undertreated	380	215	(57)	12	(3)	153	(40)
Condition untreated	127	74	(58)	1	(1)	52	(41)
M(onitoring)	**222**	**221**	**(99)**	–	–	**1**	**(1)**
Laboratory monitoring	152	152	(100)	–	–	–	–
Non-laboratory monitoring	70	69	(99)	–	–	1	(1)
E(ducation or information)	**62**	**62**	**(100)**	–	–	–	–
Disease management or advice	62	62	(100)	–	–	–	–
N(on-clinical)	**55**	**54**	**(98)**	**1**	**(2)**		
Other	55	54	(98)	1	(2)		
T(oxicity)	**165**	**145**	**(88)**	**19**	**(11)**	**1**	**(1)**
Toxicity, allergic reaction or adverse effect present	165	145	(88)	19	(11)	1	(1)
Overall	**1656**	**1348**	**(81)**	**94**	**(6)**	**214**	**(13)**

Bold entries represent the most important outcomes

*N* number

^a^These three DRPs were mistakenly coded duplicate medication by the pharmacists. DRPs were (1) codeine and oxycodone (pseudo-duplicate), (2) allopurinol and colchicine (overtreatment), and (3) two different dosages of doxazosine were in use simultaneously (dose too high)

^b^The percentage within DRP type or subtype

The majority of STOPP criteria was associated with DRP type “drug selection” (59/94, 63 %), whereas START criteria were associated with DRP type “undertreated” (205/214, 96 %). Both “undertreatment” and drug selection were more frequently identified in the absence of either STOPP or START criteria. Seventy-nine percent of the DRP type drug selection and 57 % of the DRP type undertreated did not comprise the criteria. The most common DRP type related with the recommendation to cease a drug was “no indication apparent” (186/338, 55 %). The most common DRP type related with the recommendation to add a drug was an untreated symptom that emerged from the patient interview, e.g. pain, itching or shortness of breath (32/275, 12 %).

### Implementation rate of recommendations associated with STOPP/START criteria

Thirty-five percent of DRPs were associated with a recommendation to cease, replace or add a drug. The implementation rate for recommendations associated with STOPP criteria was higher compared to recommendations associated with START criteria (56 vs. 39 %, *p* = 0.005). Both implementation rates of recommendations associated with STOPP and START criteria were lower compared to recommendations not associated with STOPP or START criteria (66 %, *p* = 0.047 and *p* < 0.001, respectively, Table 3).

**Table 3:** Comparison of prevalence and implementation rate of recommendations to stop, add or replace a drug, associated with STOPP/START criteria and implicit criteria

Type of recommendation	No STOPP/START			STOPP		START		*P* value
	*N*		IR	*N*	IR	*N*	IR	
Cessation of drug	**259**	**51** %		79	58 %	–	–	0.23
Addition of a drug	**78**	**54** %		–	–	197	38 %	0.02
Replacement of drug	**138**	**51** %						
				15	47 %	–	–	0.75
				–	–	17	53 %	0.83
Other	**873**	**75** %		–	–	–	–	–
	**1348**	**66** %						
Total				94	56 %	–	–	0.047
				–	–	214	39 %	<0.001

Bold entries represent the most important outcomes

*N* number, *IR* implementation rate

STOPP criteria were applicable to 79 of 338 recommendations to cease a drug (23 %). The implementation rate for STOPP criteria was not different compared to other recommendations to cease a drug (58 vs. 51 %, *p* = 0.23). START criteria were applicable to 197 of 275 recommendations to add a drug (72 %). The implementation rate for the subgroup START criteria recommendations was lower compared to other recommendations to add a drug (38 vs. 54 %, *p* = 0.02) (Table 3).

### Prevalence and types of STOPP/START criteria

STOPP criteria were present in 80 patients (17 %). Sixty-nine patients had one potentially inappropriate drug, and 11 had more than one. START criteria were present in 163 patients (36 %). One hundred twenty-two patients had one potential prescribing omission and 41 had more than one.

Nine types of STOPP criteria accounted for 82 % of the total and 25 of the 65 available types of STOPP criteria were present. The most prevalent STOPP criteria were duplicate drug classes (*N* = 19, 20 %), benzodiazepines (*N* = 12, 13%) and vasodilator drugs (*N* = 12, 13 %) (Table 4).

**Table 4:** Implementation rates of ten most frequent potentially inappropriate medications according to STOPP criteria

Inappropriate medication	*N*	IR (%)
Any duplicate drug class prescription	19	47
Drugs that adversely affect fallers: Benzodiazepines	12	67
Drugs that adversely affect fallers: Vasodilator drugs	12	50
Long-term (i.e. >1 month), long-acting benzodiazepines	7	71
Aspirin—not indicated	6	33
Oestrogens without progestagen in patients with intact uterus	5	100
NSAID with heart failure	5	80
Long-term NSAID or colchicine for chronic treatment of gout - no contraindication to allopurinol	5	80
β-Blockers and frequent hypoglycaemic episodes	3	67
Long-term opiates in those with recurrent falls	2	50

*N* number, *IR* implementation rate

Ten START criteria accounted for 89 % of the total, and 18 of the 22 available START criteria were present. The most common START criteria were calcium and vitamin D in osteoporosis (*N* = 58, 27 %), statins in coronary, cerebral or peripheral vascular disease (*N* = 31, 14 %), and β-blockers in angina, acute MI or heart failure (*N* = 20, 9 %) (Table 5).

**Table 5:** Implementation rates of ten most frequent potential prescribing omissions according to START criteria

Omitted medication—medical condition	*N*	IR (%)
Calcium and vitamin D supplement—osteoporosis	58	57
Statin therapy—history of coronary, cerebral or peripheral vascular disease	31	26
β-Blocker—angina, acute MI or heart failure	20	15
Proton pump inhibitor—ASA (≤100 mg) and >80 years, NSAID and >70 years or reflux^a^	19	79
Bisphosphonates—corticosteroids or osteoporosis	16	13
Statin therapy—diabetes mellitus	13	23
ACE inhibitor—heart failure	9	44
Metformin—type 2 diabetes or metabolic syndrome	9	22
ACE inhibitor or angiotensin receptor blocker—diabetes with nephropathy	8	50
Antihypertensive therapy—systolic blood pressure >160 mmHg	8	25

*N* number, *IR* implementation rate

^a^This START criterion is an adapted version of the original STOPP criterion, as used in the Dutch guidelines [8]

## Discussion

This study shows that the majority (81 %) of DRPs identified by pharmacists during a clinical medication review was not associated with STOPP/START criteria. START criteria identified twice as much DRPs compared to STOPP criteria. In contrast, the implementation rate of recommendations originating from STOPP criteria was higher compared to recommendations originating from START criteria. Recommendations not originating from STOPP/START criteria, however, had a higher implementation rate than both STOPP and START criteria.

The majority of DRPs (65 %) identified during medication review is not associated with recommendations to cease, replace or add a drug, and could therefore not be detected with STOPP/START criteria. Furthermore, only half of the recommendations to cease or add a drug are associated with STOPP/START criteria in this study. In particular, only 23 % of all recommendations to cease a drug comprised a STOPP criterion. The most important reason to cease a drug was no indication apparent for a drug. The majority of DRPs can therefore only be detected by a structural assessment of the patient’s medicines and diagnoses using the implicit criteria. These findings underline the importance of using implicit criteria for medication review and education of the community pharmacist to develop the required medication review skills to use them [26].

START criteria were applicable to 36 % of patients which was considerably higher than in the study of Ryan et al. (23 %) [27]. Eighteen of 22 criteria accounted for the prescribing omissions in our study, while this was 15 in the study of Ryan [27]. The high prevalence of START criteria in our study together with the high proportion of recommendations to add a drug associated with START criteria suggests a good practical applicability of this tool for older patients with polypharmacy in primary care. Other reasons to add a drug were mainly based on the complaints of the patient that emerged from the interview. These problems are diverse and could not easily be converted into a START criterion.

The implementation rate of recommendations associated with STOPP was comparable to other recommendations to cease a drug. On the contrary, recommendations to add a new drug based on START were less frequently implemented compared to other recommendations to add a drug. Especially, recommendations to add cardiovascular drugs (e.g. statins, ACE inhibitors and beta-blockers) were poorly implemented. It is likely that GPs are cautious to change cardiovascular treatment of patients who are concurrently seeing a specialist. Furthermore, non-acceptance may be caused by the fact that patients previously experienced adverse effects on these drugs. Although these adverse effects are probably not always that serious that rechallenge is unacceptable, patients will often be reluctant to restart such drugs. Finally, GPs may be reluctant to add preventive drugs in the oldest old, because risk factors such as high cholesterol levels and hypertension for those patients may not be related to mortality [28, 29]. On the contrary, addition of proton pump inhibitors and, to a lesser extent, calcium and vitamin D had high implementation rates. These drugs are characterised by a direct effect or by the absence of serious adverse effects.

Our study had several strengths. First of all, the elaborate description of DRPs and recommendations by the pharmacists enabled retrospective identification of STOPP/START criteria. Second, we used data from routinely performed medication reviews involving a high number of community pharmacists, GPs and patients. The results are therefore likely to be representative for daily clinical practice in primary care.

There were some limitations to the study. First, we could not directly apply the STOPP/START criteria because the researchers, unlike the pharmacists, did not have access to medical records (diagnoses and laboratory values). Therefore, the study design did not allow for a direct comparison of a strategy purely based on STOPP/START criteria and a strategy based on implicit criteria. Although for a limited number of STOPP criteria, clinical information is not required, and access to the full clinical record is recommended for the majority of STOPP and START criteria [30].

Despite this, STOPP criteria in our study were applicable to 18 % of the patients, which is slightly lower than the findings of Ryan et al. in a comparable primary care population [27]. In our study, 25 of 65 STOPP criteria were used, which is comparable to the study of Ryan et al. using 28 STOPP criteria [27, 30].

Second, the pharmacists who performed the medication reviews did not have specific training in the application of STOPP and START criteria. However, the pharmacists were considered to have sufficient knowledge of the guidelines underlying these explicit criteria, based on the training programme and monthly web conferences. Still, it is likely that applying the STOPP/START criteria on the medication data of the original population selected for medication review would also have identified some DRPs that now have been missed by the pharmacist. Thirdly, the implementation rate of recommendations was based on self-report by the community pharmacists and not on measurement of medication changes in dispensing records. Finally, by including only patients taking five or more medications, bias could be introduced by potential underestimation of prescribing omissions as detected by START criteria.

Although STOPP/START criteria were present in a minority of all DRPs identified, especially, START criteria do seem applicable as a screening tool for medication review in primary care. It has been suggested to incorporate STOPP and START in existing information systems in primary care [27]. Such automated systems could facilitate medication review but cannot replace a systematic approach using implicit criteria. Finally, explicit criteria will remain susceptible to changes, which are shown by the recently published second version of the STOPP/START criteria [31]. Future research should further establish the applicability of STOPP/START criteria in medication review by incorporation of the tool into the intervention.

## Conclusion

This study shows a higher prevalence of START criteria compared to STOPP criteria in identified DRPs of community-dwelling older patients, while STOPP criteria are implemented more frequently. Although STOPP/START criteria identify an important number of DRPs, the majority of DRPs identified during medication review was not associated with STOPP/START criteria. When used, START criteria may have a higher practical applicability compared to the extensive list of STOPP criteria for medication review in primary care. These findings suggest that health care providers cannot solely depend on the STOPP/START criteria to identify DRPs in primary care.
